# Novel Application of Electro-Chemistry to Therapeutics

**Published:** 1855-04

**Authors:** 


*Novel Application of Electro-Chemistry to Therapeutics.*—Chemistry is about to save from death, or a premature old age, those artisans whom the exercise of a cruel profession condemns to breathe metallic dust or vapors, who poison themselves daily for the sake of living, and acquire so many dreadful infirmities in the silvering of looking-glasses, the preparation of white lead, or working in the mines. Science comes to the help of the victims of industry or pleasure, and extracts from their bodies, atom by atom, the devastating metal that had fastened on their tissues, and weighed on the springs of life. These hopes are drawn from a memoir presented to the Academy of Sciences by M. Dumas, and the authors of which—MM. A. Poey, of the Havana, and Maurice Vergnès—will hold a distinguished rank among the benefactors of mankind, if experience confirms their assertions.

The invention consists in the application of electro-chemistry to the cure of the diseases we have mentioned; and surely, of all its marvellous uses, this would be the most admirable.

M. Poey takes an unfortunate patient, corroded by lead, mercury, gold, silver, or any other metal, and places him in a metallic bathing tub, isolated from the ground. The man sits down, his legs horizontally stretched out on a wooden bench, isolated from the tub, which is filled with water up to his neck. The water is slightly acidulated, to increase its conductibility; and the acid varies according to the cases. Nitric or hydrochloric acid is used for the extraction of mercury, silver, or gold; sulphuric acid for that of lead. This done, the negative pole of a pile is brought into contact with the sides of the bathing-tub, and the positive pole placed in the hands of the patient.

The work of purification is now in full activity; the electrical current precipitates itself through the body of the sufferer, penetrates into the depth of his bones, pursues in all the tissues every particle of metal, seizes it, restores its primitive form, and, chasing it out of the organism, deposits it on the sides of the tub, where it becomes apparent to the naked eye.

In this great discovery, chance or accident has played a part. One of the inventors—M. Maurice Vergnès—occupied himself with galvanic gilding and silvering. His hands being in continual contact with solutions of nitrate and cyanuret of gold and silver, got covered with ulcers in consequence of the introduction of metallic particles. One day he plunged the diseased organs into the electro-chemical bath, at the positive pole of the pile; and, after a quarter of an hour, to the great surprise of the beholders, a small plate of metal brought into contact with the negative pole covered itself with a thin coating of gold and silver, extracted from the hands of the operator, whence the most powerful remedies had not been able to eliminate them. This discovery was made on the 16th of April, 1852.

The authors employ a pile of thirty pair of plates, approaching, at the same time, that of Bunsen and of Grove, as coke and platina enter into its composition, by which its action is rendered more energetic. Each pair has a diameter of 40 millimetres, and is 217 millimetres high. The number of the pairs to be used at the beginning of the operation depends upon the temperament of the patient and the nature of the malady. Thus, a delicate and very nervous person is at first submitted to the action of ten or twelve pairs only, and every five minutes the number is increased. A person of a sanguine or lymphatic temperament is able to endure a greater number of elements. The same observation applies to the quantity of acid employed in the bath, less being required for a nervous than a lymphatic constitution.

The metallic atoms extracted from the body deposit themselves on the whole surface of the tub; but they are more abundant opposite to the part of the body where the metal was lodged. The size of the metallic spots varies considerably; some are microscopical; others have the dimensions of a pea; those of the size of a pin's head are very common. "I have seen," says M. Poey, "after the bath of a person who complained of pains in the arms, from having taken mercury, the contour of the arm perfectly drawn upon the metallic plate by the deposit of metallic atoms that without doubt proceeded from the suffering member."

We shall terminate our article with an experiment made before the members of the Faculty of Medicine of the Havana.

A patient had undergone during a whole week an external mercurial treatment (frictions, with mercurial ointment.) He had then taken several lukewarm baths, and it could not be supposed that any mercury still remained attached to the skin.

He was put into a water bath mixed with muriatic acid. After having remained in it for five minutes, some of the water was taken out, and afterwards analyzed by M. Baraceca, who found no traces of mercury in it.

The circuit was then closed; and, after the electric current had acted for about an hour, a new sample of the water was taken. Mixed with an alkaline sulphuret, the water became black; and a piece of copper having been dipped into it, gave sure signs of the existence of a small quantity of mercury. Thus the water of the bath now held mercury in solution.

During the experiment, a perfectly clean piece of copper had been placed at the negative pole. When it was taken out of the water, towards the end of the operation, its yellow-greenish color not only testified an oxidation in which mercury had taken a part, but small white spots were scattered over the surface, one of which, of the size of a square line, was very brilliant and of a mercurial whiteness. The plate having been heated underneath, the spot disappeared, and the original color of the copper was restored, which proves that the spot was mercurial.—*London Med. Times and Gaz.*, from *La Presse.*

